# Transactions of the American Medical Association

**Published:** 1848-12

**Authors:** 


*Transactions of the American Medical Association.* Vol. 1, 1848, pp. 403, 8vo. Philadelphia: Printed for the Association.

It was with no small emotion that we began the perusal of the first volume of the Transactions of an Association, which, by its title, composition, and heralded aim, is meant to represent the medical profession in the United States. If we have been less sanguine of the successful results of these combined efforts, we should say rather of the intention to secure combined efforts, than some of our friends, it is not because we undervalue the importance of the objects, or the zeal and ability of those enlisted for their attainment; but because of inherent difficulties, to overcome which, demands a matured plan, a good deal of experience, and a prudent spirit of compromise as well as of conciliation. Influenced by these considerations, we cannot be supposed to join in the *io pæan* for a triumph not yet achieved, nor, on the other hand, to examine in an over critical mood the officially recorded acts of the new association.

The first and second conventions, held in New York in 1846, and in Philadelphia in 1847, from which originated the American Medical Association, were occupied with preliminary measures of organization, and the highly important subjects of ethics and medical education. In the main, a tolerably general, and, in some instances, a decidedly cordial assent to the recommendations on these subjects, has been given by the profession throughout the United States. Of their entire appositeness and wisdom, we have expressed some doubts on former occasions, in this journal, and we may hereafter feel ourselves called upon to offer farther dissent. This will be, however, more against extreme views than against ameliorations and improvements, the utility of which we would be slow to contest. For the present, our attention will be more particularly directed to the reports of the several committees on *Practical Medicine*, *Surgery*, and *Obstetrics*, and on *Medical Sciences* and *Medical Literature*, made at the meeting of the association in Baltimore, in compliance with the terms of their appointment the preceding year in Philadelphia. These committees consisted, each, of five members; of whom three resided in the same place. It was thought that, in this way, the services of a majority of each committee would be secured for ready conference and conjoint labour; while the assistance of the remoter members could be obtained, by their written advice or contribution towards the elucidation of a designated subject. Good as was the scheme in theory, its actual workings have not, we fear, corresponded with the design of the association in framing it. For the most part, the reports, so far, have been the work of the chairmen of the respective committees, unless the gentleman first named on the list has been prevented by unforeseen obstacles, from applying himself to the assigned task. It may have happened, in the case of some of the reports of a date antecedent to those contained in the present volume, that the chairman who had industriously collected and arranged materials for the report, with the apparent concurrence of his immediate associates, has been suddenly tripped up, and his homogeneous collection replaced by hasty gatherings from various quarters, including some from his own store. But the point to which we would more particularly advert, just now, is, that the reports in the volume before us are, mostly, the work of individuals, and not of the collected intelligence and learning of the committees. We make this remark in justice both to the members composing the latter, and to the reporters themselves; and not with a design to censure, or to offer a substitute for the present system, which, it should be remembered, is the one pursued in nearly all deliberative assemblies. Perhaps misconception, at a distance, would be prevented by a general imitation of the practice of the Philadelphia College of Physicians, which designates one member to report annually on a branch of medical science. An awkward feature in the common plan of committee reports is, that the members sometimes affix their signature to an exhibition of facts and opinions to which they have barely directed their attention, certainly have not studied them in their chief bearings. Occasionally, too, a direct contradiction is seen between the views and criticisms advanced in the report, and those antecedently held and promulgated by some of the signers. Of this we shall offer an example, at least, when we come to notice specifically the reports in the present volume.

The embarrassment unavoidably growing out of the difficulty, not to say impossibility of assigning fixed limits to the several divisions of medicine, including both its science and its practice, is apparent in the reports presented to the association. Thus, for example, Dr. Wragg, in reporting on "Medical Sciences," and Dr. Joseph M. Smith on "Practical Medicine," meet on the field of pathology and therapeutics—as on ground common to both of them. Of more importance to the reader is the pleasant reflection, that each of these gentlemen has brought together a valuable array of facts and conclusions, well calculated to advance the cause both of medical science and of medical practice. In Dr. Wragg's neat summary, "Hygiène" has not received the proportionate share of notice to which its vast importance entitles it. In this we but find, we are sorry to say, an indication of the too general indifference with which the whole subject is regarded by the great body of the profession, wherever the English language is spoken.

Dr. Smith, in his etiological inquiries, transports us from the practice into the theory of medicine, but in a manner which we can scarcely blame, so gentle is the transition. His chief theme being Epidemics, it was difficult to avoid the course which he has taken. We cannot praise his division of epidemics into *Contagious*, *Infectious* and *Meteoratious*, even if we were more assured of the last word being pure English, and framed in conformity with the analogy in such cases. The substantive, *meteoration*, also used by him, sounds harshly to our ears. The chief part of the report by Dr. Smith is devoted to typhus fever, and its chief ally, if not other self, typhoid fever, for the study of which, the vast number of emigrants from Europe, arriving in New York and other cities on the Atlantic sea-board, who were victims to the disease, have furnished ample materials. Dr. Smith believes that the facts which he enumerates, "fully warrant the inference, that the diffusion of typhus is favoured by a peculiar ærial influence which occurs periodically. Such an influence is sometimes widely extended." He tells us: "But although the disease among residents has, in most instances, been traceable to exposure to the poison, emanating from the persons and effects of emigrants, there is reason to believe that it has, in some instances, originated *de novo* in the close and sordid dwellings of the poor in our large seaport towns; and that the mortality from the disease has been augmented from such sources."

Dr. Smith proclaims his conviction of the identity of typhus and typhoid fever, for, as he tells us, "every where (in the report) have the two forms of disease been associated and looked upon as one and the same malady." He confirms this proposition in a review of the symptomatology and morbid anatomy of typhus and typhoid fever; and by a citation of cases which imparts the force of conviction to our minds.

Appended to this report is a paper, of no small practical importance, on *Œdematous Laryngitis*, by Gurdon Buck, Jr. M. D., Surgeon to the New York Hospital. The object of the writer is to show the success with which this ever alarming and so often fatal disease has been treated by means of "scarifications of the glottis and epiglottis." Four plates are introduced; two exhibiting the morbid condition of the parts in cases of typhus, another a view of them and the manner in which they are reached in the operation, and a fourth of the instruments used for the latter purpose.

The "Report of the Committee on Surgery," consists of two parts; the first read by Dr. Norris, the second by Dr. Parrish. The chief topics in the former, are lithotomy and lithotrity, the treatment of aneurism by compression, of hydrocele and other serous effusions by iodine injections, and of fractures of the condyles of the *os humeri*. The Reporter concludes with directing attention "to derangement of the cerebral functions, following ligature of the common carotid artery." Dr. Parrish read the portion of the report on "Anæsthetic Agents," in which (including an appendix) he embodies a large number of cases and conclusions on the highly important, but still unsettled question, of the real value of these agents in surgery. Dr. H. J. Bigelow supplied the first paper in this appendix, under the title of "Anæsthetic Agents, their mode of Exhibition and Physiological Effects," followed by a "List of Patients, who have inhaled ether or chloroform for surgical operations in the Massachusetts General Hospital up to April 1st, 1848." A similar table has been furnished by Dr. Watson for the First and Second Surgical Divisions of the New York Hospital. Dr. H. H. Smith and Dr. Mütter report the cases in which these agents were used in the clinics of the University of Pennsylvania and of the Jefferson Medical College. Dr. Mussey, in a letter, communicates the results of his experience in twenty-two operations.

The "Report of the Committee on Obstetrics," made by Dr. Harvey Lindsly, consists, with two exceptions, of a brief summary of the utility, and conditions for the use of anæsthetic agents in the practice of midwifery. The exceptions are: "The use of ice to promote uterine contractions," and of the roots of the cotton plant for the same purpose. Without meaning to detract from the importance of the topic selected, we cannot but believe that a much wider range of inquiry was demanded of the reporter, so that he might have done more justice to the department of Obstetrics, which presents so many aspects of interest to the practitioner.

The "Report of the Committee on Education," was read by Dr. Wellford. Every member took a part in the preparation of the report, as we learn from a note at the end of it; but "the chairman (Dr. Stevens,) takes occasion to offer his thanks to Dr. Pliny Earle, to whom it chiefly owes its present form." In addition to repeating the recommendations of the committees on preliminary education, and on the requisites for graduation, submitted to the Medical Convention which assembled in Philadelphia, the present one urges, in the shape of resolutions, the importance of attention to sundry observances on the part of the faculties of medical schools and the trustees of hospitals, for rendering medical instruction, more demonstrative and clinical. These resolutions and other parts of the report will find a place in our pages, accompanied, most probably, by some commentaries on the feasibility and value of the reforms recommended.

The "*Report of the Committee on Medical Literature*," falls short of the requirements of the subject. Dr. Holmes relieves, it is true, the tediousness necessarily arising from an enumeration of the titles of works, by lively sallies and sparkling epithets; but he scarcely goes below the surface. He culls here a bud, there a flower; but we miss the matured fruit, nor is the necessary culture for the largest product pointed out. In vain will the student look for a knowledge of the beginning and progress of our medical literature; and of the circumstances which might favor its vigorous development. Something more than a lively imagination and a hurried glance over the titles of books and journals is required for the performance of the task assigned, for the nonce, to the committee of the American Medical Association, and in this instance undertaken by the chairman, Dr. Holmes. This gentleman winds up his report by indicating what he believes to be the obstacles to the attainment of an independent medical literature, and, also, the signs—we cannot call them reasons—why there should be such independence in our medical investigations. But we will give his own language on the occasion, as follows:

"Two fatal influences have acted not merely on medical science, but on all natural science in this country. The first is the habit of indolence generated by the easy acquisition of a foreign literature which seems to answer every necessary purpose. The second is the habit of negligence which springs from the curious fact of a constant parallelism, which is not identity, in most natural objects and phenomena of the New World, with something of the older continent. In literature this has enfeebled the relation between words and realities; in science it has induced the same laxity and incoherence. The American constitution must be studied by itself—it differs from the European in outline, in proportions, in the obvious character of the skin and hair—why should it not differ in the susceptibilities which, awakened, become disease? The American climate remoulds the European, and casts a new die of humanity—will it not generate causes of disease different from those of the Old World? Over this virgin soil a new Flora is waving her long web of tapestry, flowing from the lichens of Katahdin to the myrtles of Cape Sable; is there no undiscovered healing in any of its leafy and blossoming folds? Here is the true field for the American medical intellect; not to set English portraits of disease in American frames; not to trust for immortality to a little more or less of manual adroitness or questionable hardihood; but to co-operate with that fast-gathering band of students who, in other departments of science, are studying what nature has done with her American elements, and teach us what disease is here, how it is generated, and what kindly antidotes have been sown in the same furrows with its fatal seeds."

Pleasant reading is this same passage of Dr. Holmes—poetical, and not wanting in terseness withal; but of its logic we have some doubts. Room is not allowed us to engage in the general argument, as to the real influence and effects of the literature of England on the American mind. It is barely possible that, by this time, we might possess more original poets and essayists, if all intercourse had been cut off between the Pilgrim Fathers, to the north, and the cavalier gentry to the south, and their father-land. We might, perhaps, have a more fully described Flora, and a more salient Zoology. It is not, however, by any means clear that we should possess as many readers, as widely diffused and as much general knowledge, and as many incentives, in the way of suggestion and example, to all kinds of intellectual labour, as now characterize our people. Our language might exhibit the pedantry of the first James, or abound in the euphuisms of the Elizabethean age, but it would scarcely exhibit the copiousness and flexibility which now belong to it. It may be alleged that, in this as in the various departments of literature, scholastic and imaginative, there would have been here, as there has in England, a gradual development, until something like a distinctive and national character was attained. We cannot forget, however, our peculiar position and wants, nor how much of our time and energies, during all this period, from the first embarkation down to the present day, has been expended on the material world; and how little was allowed for our attaining profound scholarship, or opening new sources of invention and imagination. If we had been cut off from English literature and science during all this time, would our colleges be now more numerous and better endowed; our press more vigorous and diversified in its manifestations; the intelligence of the people of a higher standard; the useful arts richer in application, and the fine arts have enlisted more genius and gratified more tastes? We do not believe they would. On the contrary, deprived of much varied and substantial aliment, as well as of allowable condiments, from abroad, the national mind would have suffered in consequence, if not from positive inanition, at any rate from defective and irregular functional exercise—coarse excitement alternating with apathy and languor—the condition, in a somewhat better degree, of the aboriginal Indian. Besides, the question is complicated by the fact, that opportunity could not be given for the evolution and augmentation of American literature, unaffected by European immigration, and especially of immigrants speaking the English language, even if literary non-intercourse had prevailed. Most wisely, and for us, happily, has it been ordained, that, during all the time in which Europe has been sending so many of her ignorant and destitute inhabitants to the United States, she has, also, sent a perennial supply of the intellectual productions of her chosen sons, whose treasures of historic and philosophic lore, and beautiful imaginings in poetry and the arts of design, should be regarded as an equivalent to the expenses to which their fellow-countrymen may, from time to time, have subjected us, as well as a corrective of the more serious evils of moral and intellectual deterioration.

Dr. Holmes overrates the influence of "the habits of indolence generated by the easy acquisition of a foreign literature which seems to answer every necessary purpose," in qualifying it as a fatal one. If a literature be rich and productive, the easy terms on which it is acquired can hardly stand in the way of its usefulness. Men are not prone to undervalue the knowledge procured by genius with scarcely an effort; or to aver that it is deleterious to the possessor, and that its healthy and advantageous use depends on the slowness and difficulty with which it is attained One might be led to infer from the strain of Dr. Holmes' remarks, that the American people are suffering from cerebral dyspepsia, the result of inordinate cramming of the intellect with English literature and science, in as fearful a manner as their gastric functions and nervous system, generally, are deranged by ardent spirits and tobacco. Would his fears lead him to advocate a high tariff on the productions of English mind, similar to that which many have thought requisite on the productions of English machinery and handicraft ? Or, would he imitate the more exclusive patriot, and refuse to clothe himself in English ideas or modes of thought, and abjure English ornaments of speech, whether in poetry or oratory ?

Such a view as that advanced in the report not only fails to represent the actual condition of medical science among us, but it does injustice to the American mind ; the extreme activity and wide grasp, not to say restless energy and unceasing adventures of which, prove any thing rather than indolence or the apathy that may be supposed to result from a too ready and excessive repletion. When it speaks through the press, in journals, or separate essays, or from the professor's chair, either in his commemorative discourses or in his regular lectures, do we find it manacled by foreign technicalities, and oppressed by lore, either ancient or modern ? We opine not. It is greedy for literary aliment ; but in general, for reasons already assigned, it has neither the time nor the opportunity for a very nice selection and deliberate appropriation. It suffers more from defect than excess of supply ; from a difficult rather than from an easy acquisition. Ask the practitioners of medicine, both in town and country, spread over the vast area of the United States, whether they have fallen into habits of indolence from a too easy acquisition of English medical science, and from having over-read; and whether their patriotism and pride are hurt by being invited to look at " English portraits of disease in American frames ?" We rather think that the answer will be : " Situated as we are, drudges in the daily and often nightly routine of hurrying and exhausting occupation, with little time to contribute ourselves, and with small expectation of much methodical contribution from our associates, rather than look at bare walls, or content ourselves with a few rough home sketches, we will take, without grudging, English portraits of disease in American frames. We will not find fault with those who made the frames, because they did not paint the portraits."

The figurative style of rebuke in the words to be now quoted from the report, is meant to apply to the system of editing the works of British authors, which is designated as "the great *forte* of American medical scholarship." Some of its abuses are pointed out in the following :

"Sometimes the additions by the 'American Editor' have been real and important, oftener nominal and insignificant. The following calculation of the proportion added to different recently published works, taken at random, will show the average amount of material so contributed. The Editor's proportion was, in two instances, one fourth; in two more one-eighth; in one one-ninth : in another one-tenth ; in others one-fifteenth, one-seventeenth, one-nineteenth, one-twentieth, one-twenty-eighth, one-fifty-ninth, one-sixty-fifth, one-ninetieth, one hundred and seventh, and, in one instance, such a sprinkling as a single penful of ink might furnish, and leave enough to spare for a flourishing autograph. The fairest fruits of British genius and research are shaken into the lap of the American student, and the great danger seems to be, that in place of the genuine culture of our own fields, the creative energy of the country shall manifest itself in generating a race of *curculios* to revel in voracious indolence upon the products of a foreign soil!"

The point of this amusing sarcasm is not at all blunted by the fact, that the reporter, Dr. Holmes, did, himself, some ten or twelve years ago, in conjunction with his friend, Dr. Bigelow, figure in the capacity of editor of Dr. Marshall Hall's work on the Practice of Medicine. Whether the want of a second call for his editorial services on this book has quickened the perceptions of Dr. Holmes to the vanity and bad effects of the practice, we will not take upon ourselves to say. But, our friend Dr. Bell is one of the signers of this report, and so far, formally, an approver of the ridicule cast on the poor editors. He has no cause of complaint, that his labours have not been well received. It may be, that both he and the Chairman of the report, like true penitents, seeing the error of their ways, have agreed to inflict on themselves, as penance, this public chastisement, in lieu of stripes. We shall not be surprised, after this, to learn that Dr. Bell has detached himself from the partnership with Dr. Stokes, and has determined, on the next occasion that offers, to set up for himself.

On our part, however, we are not disposed to recommend an abandonment of the practice of editing, because it has been abused. Dr. Holmes's reading must have supplied him with abundant examples of it, in the literature of other nations, and of its having been undertaken by men of unquestionable ability, and not lacking, when the case required it, originality. Dr. Bell will allow us to make use of some of the references made by him in his *Bulletin of Medical Science*, August, 1846, on this topic.

"Reverting to medical literature, and coming near home, our friend Dr. Meigs of the Jefferson College will not, we are sure, accuse us of uncalled for personality, if we assert that he was not the less able and willing to write his own work on Midwifery, after having translated and edited that of Velpeau; nor did he think that he was attaching himself, as a parasite, to a work of merit issuing from the French press, when, owing to his want of time to prepare an original volume on the Diseases of Females, he translated that of Colombat, and enriched it with a large body of notes. South, the translator and copious commentator of the Surgery of Chelius, now in its passage through the press, is not twitted for his servile labour; but, on the contrary, receives from his countrymeu all praise for his industry and talent. Dr. Gross, in the early period of his professional career, in this city, was known and esteemed for his translations from the French and German; and, afterwards, he did not think it beneath him to edit Liston's Elements of Surgery, although, in the meantime, he had brought out his elaborate work on Pathological Anatomy. We are sure that he does not regret his exercises as translator and commentator, nor think that they interfered with the originality of his work on 'Wounds of the Intestines,' any more than that they have weakened him for his present career as a successful surgeon and able lecturer."

Continuing the examples thus pertinently introduced, we would cite the experience of Dr. Condie, whose long previous labours, as translator, commentator and journalist, do not seem to have disqualified him for writing his excellent and approved treatise on the Diseases of Children. The immediate precursor of this work, was his edition of Evanson and Maunsell, on the same subject. So, also, the conjunction of the Clinical Lectures of Dr. Gerhard with those of Dr. Graves cannot be adduced in proof of any conscious inferiority or feeling of literary dependence, on the part of the American associate. That the practice which we are now defending, is not entirely unknown in Great Britain, appears in the last Dublin edition of Dr. Graves' Lectures, which are brought out, we observe, under the editorial supervision of Dr. Neligan. This last named gentleman does not seem to be under any apprehension of forfeiting his previous standing, as the author of a "Treatise on Medicines," or of being thought parasitical, or classed with the *curculio* tribe, by his professional brethren.

The question may be stated in another way. A physician in place of writing a slim octavo, containing the result of his observations and study of a particular disease, or a class or series of diseases, may deem it advisable, at the suggestion of a publisher, to distribute his materials, in the shape of notes or commentaries, through a work by another writer. If he appear before the public in the first case, with his name alone on the title page, he is called the author of an original work. But as editor, he becomes merely a parasite, *curculio*, &c., in the opinion of certain critics. His medical brethren who read, do not, however, join in these sentiments. They prefer, and this publishers who feel the public pulse well know, edited works. A volume on Materia Medica is not deteriorated in their eyes, by its being a translation from the French or German, or by its having notes and appendix by an "American Editor," in which the virtues of our indigenous plants are set forth, and their modes of preparation and other matters in the American pharmacopœia detailed. So, also, in the pathology and treatment of disease by an English or a continental writer, a corrective of extreme views or of a fashion of practice not adapted to our climate, and contradicted by home experience, may be introduced in notes. Even in its literary aspect, an additional case, an emendation of date, a new reference, can hardly be called intrusive or superfluous—especially when we bear in mind the circumstances of the great body of medical readers, who have not the time for comparing and collating among their own literary stores, even if these were more ample than they usually are.

We must believe that the late Dr. Rush was of this opinion, when he introduced to the American profession the writers whom he most prized, with notes, in the way of addition and commentary. He, who signed the declaration of political independence, and who was as desirous as man could be, to throw off all the technical trammels of the European schools, did not deem his patriotism tarnished, or his own professional dignity abated, by his becoming an "American Editor," or by his setting "English portraits of disease in American frames.' If it were necessary to enforce farther the argument, it would be easy to show, that the fashion of editing is common enough in works on theology,* polemical divinity, and law. Let, then, our reviewers and reporters of committees confine their critical and censorial duties to an inquiry into the manner in which the task of editing is performed, in each particular case in which it is brought to their notice; but not indulge in wholesale and indiscriminate censures of the practice in general, which are both illiberal, and, so far as they are opposed to literary progress, illiterate.

In commenting on the main points of the report of Dr. Holmes, we bear in mind his position as teacher and author in the profession, to say nothing of an enviable fame acquired in other departments of literature. These justify a freedom of remark which would be misspent on inferior minds; but which he can, we believe, not only tolerate, but relish, as it evinces some of that independence of opinion and judgment, which he himself seldom withholds when demanded by the occasion. We must not conclude, however, without apprising our readers of the divisions of his report, and the scope of his narrative, for such it is, in a great degree. The committee, in taking a summary retrospect of what has been done in the several branches of medicine, have saved their successors much trouble, and the tediousness of the long array of names which, in the present report, have been unavoidably enumerated. This should be taken into account, in forming an opinion of the labours of the reporter on the present occasion. He must have felt an embarrassment, in framing a philosophic view and exhibiting distinctive traits among the successive crowds of titles of books and essays, similar to that of which Boileau so feelingly complained, when he wished to reduce to poetic measure and rhyme the impracticable names of Dutch towns and rivers, in his ode to the praises of the great king, on the occasion of the campaign of the latter in Holland.

* We have just now before us the title of a work, which runs as follows: "Contemplations of Sacred History—altered from the works of Bishop Hall. By the Rev. George Henry Glasse." No threat of excommunication, no injunction, no sneers of lofty disdain, followed this well-meant labour of Mr. Glasse, which went to the length even of alterations of the original work.

"Periodical Medical Publications in the United States," is the first division of the Report. The merit of the plan of the first medical and periodical publication which appeared in this country, is awarded to Dr. Elihu H. Smith, of New York. He associated in his enterprise Dr. Edward Miller and Dr. Samuel L. Mitchell; and in August, 1797, the first number of the new journal appeared, under the name of the "*New York Medical Repository.*" Next in order of time, was the *Philadelphia Medical and Physical Journal*, 1804. "The New England Journal of Medicine and Surgery," was commenced at Boston, in the year 1812, and continued until 1826. In enumerating many of the papers contributed to the different Journals, those by one of the committee are omitted. We do not see the necessity of the committee being subjected to a self-denying ordinance of this kind. Among the numerous contributions to Dr. Chapman's Journal, in essays and reviews, by Dr. Bell, the paper of the latter on *Miasm* fairly belongs to our medical literature, both on account of the importance of the subject and of the novel view taken by the author; one deemed at the time very heterodox, but to which the tendency of later inquiries gives countenance. So, also, we miss, in the list of papers published in the "North American Medical and Surgical Journal," those in two successive numbers, by Drs. Mitchell and Bell, on the Small Pox and Varioloid Epidemic, which prevailed in Philadelphia in the years 1823 and 1824. They consist of a meteorological register, a description of variola and varioloid, illustrated with cases and *post mortem* details, and statistical returns, together with an appreciation of the value of vaccination, in a fashion which would not be unacceptable even to the Louis school. These papers were fully noticed and largely copied, in the European Journals at the time; and, if our memory serves us, a translation of them entire was inserted in the *Journal des Progres*, &c. As it was the express design of the committee to specify the Journals which had been, as well as those which are now being published, we marvel at their entire silence respecting the *American Medical Intelligencer*, which was brought out conjointly with the *Medical Library*, under the editorial direction of Dr. Dunglison, as was, at first, the *Eclectic Journal of Medicine*, and afterwards the *Bulletin of Medical Science* with the *Medical Library*, edited by Dr. Bell. A like omission occurs in the case of Dr. G.

S. Pattison's Library and Journal, of antecedent date, published in Washington by Mr. Duff Green.

When describing the general plan of division of subjects in the medical Journals, the reporter indulges in a few piquant sallies on the distribution of printed puff to the several expectants—importunate friends, dull dignitaries, and actual or expected Mecænases—and on the repetition, in Journal after Journal, of the same article. "The ring of editors sit on each other's laps, with perfect propriety, and great convenience, it is true, but with a wonderful saving in the article of furniture." A disclaimer immediately follows against any intention "to undervalue the great amount of intelligence and industry embodied in these periodicals, or to make any return of ingratitude to the faithful servants of science and humanity, who, in the midst of innumerable distractions, and often at an absolute sacrifice of their material interests, are giving their time and health, and substance, to the demands of the most exhausting department of mental labour." To this we of course must respond in terms and tone of hearty assent.

In connection with the subject of periodical literature, the reports of societies and of public institutions are introduced by Dr. Holmes. He speaks in terms of deserved commendation of the Transactions of the College of Physicians of Philadelphia; but he is at fault when, among the reports read to that body, he specifies one on Cholera by Drs. Bell and Condie. These gentlemen are authors of a small volume on this disease, which was published in 1832, and reached, in a few months, a second edition. The fact may perhaps have transpired, that Dr. Bell, as a member of a committee appointed by the College, prepared a report on Cholera, which was published in pamphlet form, a short time before the appearance of the disease in Philadelphia. Favorable notice is taken of papers published by the Massachusetts Medical Society, and of the Transactions of the State Medical Society of New York. Similar praise is due to the Transactions of the Medical Society of Tennessee, the mention of which was forgotten by the Reporter. Next, we are referred to the numerous reports from the Insane Asylums which, of late years, have been growing up in many States of the Union. "The character of the men who have been selected to manage these institutions, reflects the highest honor on the medical profession which furnished them. It would be difficult to find any body of public servants, combining a greater amount of benevolent spirit and intellectual capacity than the superintendents of these asylums." High praise this; but not beyond the merits of the parties to whom it is awarded. Then follows a brief enumeration of the more important articles which have appeared in the Journals, within the year preceding the report. We need not repeat the list here, nor the remarks interspersed through it, which are, however, in the main just and well expressed.

"In proceeding to the second division of their task, the Committee have thought it expedient, as in the portion just finished, to take a retrospective glance at the prominent works which have been published in this country in the several departments of Medical Science. They cannot pretend to give a complete catalogue, nor to criticise or characterize all the works they mention. For the sake of convenience, these works have been arranged under general heads, the first place being usually given to original productions and compilations by American authors, the second to translations from other languages, and the third to re-prints of books written or translated elsewhere." After this, we are introduced to the authors of numerous works, under the several heads of Anatomy, Physiology, Surgery, Theory and Practice. "Among the American medical authors" who "have written express treatises on the yellow fever," we find the name of Carey, who is promoted to this distinguished company, on the authority of Dr. Bartlett. The brief narrative of Mr. Mathew Carey, conveys some details and impressions which are not without interest; but, as the work of a gentleman who did not belong to the profession, and who made no pretensions to medical knowledge, we cannot understand how it should come under the head of "express treatises" on Yellow Fever. Is there not some covert satire in the Reporter's introducing a Mr. Halsted's "Account of the New Method of curing Dyspepsia," as so prominently representing the *Diseases of the Digestive Functions* in American Medical Literature? It is the only work whose title is given under this head. Some curiosity and no little credulity were displayed at the time, in New York and Philadelphia, when Mr. Halsted, at first as an amateur, and afterwards as the vender of a secret remedy for dyspepsia, promised cures to the afflicted—especially, he ought to have said, to those endowed with a lively imagination. The publication of the method of treatment, soon, however, dispelled the charm of Mr. Halsted's book, which is now, for the first time, presented as a part and specimen of American medical literature.

Dr. Clymer is mentioned in the report, with Willis and Bird, as having composed a volume on Urinary Diseases. We believe, that like some others of our friends whom we wot of, the chief evidence as yet accessible to the reader, is the publisher's announcement of Dr. C.'s intention to write such a work. The same remark applies to the lectures of Baillarger on Insanity, enumerated, in the report, among the works on this subject republished in the United States. We were not aware, before now, that our friend Warrington, so praiseworthy for his indefatigable efforts to instruct students, had written a Treatise on Midwifery, which is placed in the report on the same line with the productions of Bard, Dewees, Meigs and Tucker, as contributing, with them, to "the library of American authors"—on obstetrics. His Catechism of Midwifery comes under another category, with the "Manual of Ludlow," the "Medical Student" of Dunglison, and the Vade-Mecum of Mendenhall. Probably in the hurry of transcribing, the name of Churchill was omitted in the list of British authors on Midwifery, whose works "have been appropriated and sometimes improved by successive generations of editors." The omission is the more to be regretted, as Churchill's is a work of admitted excellence, to say nothing of the additions made by the American editor: so acceptable, indeed, has it been to our countrymen, that it has passed through three American editions in a little more than as many years.

The name of Dr. Dunglison ought to have been included among the authors of systematic treatises on *Materia Medica;* and to have displaced that of "Carpenter." We do not know of an American Dr. Carpenter, who has written on the Materia Medica, in a professional sense and for the perusal of the profession. If the Reporter had thought it worth while to notice the subject of Therapeutics in connection with Materia Medica, he would scarcely have placed on the same line, and without any discriminating remark, the compiler of a Medical Formulary, or of a druggist and apothecary's Manual, with the author of an elaborate work on Materia Medica and Therapeutics. To this error, of regarding as unit in plan and general outlines works differing from each other in many important particulars, may be traced the remark of the

Reporter: "The works of Barton, Bigelow, Coxe, Chapman and Eberle, have been, to a great extent, replaced by those of Wood and Bache, the well deserved popularity of which is still continued." The merits of this last mentioned production rest on a firmer foundation than its alleged replacing, that of Chapman or of Eberle, either of which is dissimilar to, and, of course, not replaceable by it. The same remark applies to the works (on Medical Botany) of Barton (Wm. P. C.) and Bigelow. The name merely of "Barton" fails to convey a knowledge of there having been two writers thus called, in the present category; viz., Dr. Benjamin Smith Barton, and his nephew, Dr. Wm. P. C. Barton. The former is author of "Collections for an Essay towards a Materia Medica of the United States;" the latter of a work on Medical Botany. Under this last title come "the illustrated editions of Griffith and Carson."

In speaking of the writers on the *Diseases of Women*, reference is made to a volume of letters, "by the same hand to which the translation of Colombat was owing." We do not see any prior notice of the treatise of Colombat, and the reader, not before cognizant of the fact, would not know that the translator of the French work, and the author of the lectures, is our friend Dr. C. D. Meigs.

"Introductory Lectures" and "Theses" are made, quite appropriately, themes for well grounded remarks by the Reporter. Prizes for the best dssertations upon certain specified subjects, is another feature which is also recorded. Dr. Holmes's modesty prevented him from informing us, that he had, himself, won two Boylston prizes, for Dissertations on *Intermittent Fever* and on *Neuralgia*. The critical comments upon the character and tendency of American Medical Literature are brief and pointed. Those on periodical literature are appropriate, as far as they go. From the opinions respecting the alleged obstacles to the progress of medical science in general, we have already offered our dissent, and we shall not now resume the argument.

Dr. Holmes bears rather hard on the editors of journals, for the numerous typographical blunders by which these works are disfigured. He could not anticipate at the time when he wrote the report, that it, also, would be marred in a similar manner; a result the less to be expected, as ample time was allowed, in the slow passage of the Transactions through the press, for the exercise of searching microscopical vision by the Publishing Committee. The first example, and a vexatious one, that presents itself to us, is the substitution of "Stevens" for Stearns, when it is said (page 250) of a letter in the eleventh volume of the New York Medical Repository, that it "introduced to the world the wonderful properties of Ergot." So far as the first volume of Transactions of the American Medical Association may be supposed to circulate and to acquire the weight of authority, will its readers, and especially those on the other side of the Atlantic, be misled, and Dr. Stearns to the same extent be deprived of his merited distinction. Of less moment are the following: "which" for whom, when speaking of Dr. Miller, as "one of the most original thinkers and observers which this country has produced," (p. 250) and "four first" for first four, (p. 287.)

The next *Report* is *of the Committee on Publication*, followed by the *Code of Ethics of the Philadelphia College of Pharmacy; A Statement in Relation to the United States Naval Medical Corps; A Communication on Hygiene from the Medical Department of the National Medical Institute; A Statement relative to the Extent to which Drugs are falsified, particularly with a view to their sale in the United States; A Memorial to the Senate and House of Representatives in Congress assembled; An Act to prevent the Importation of Adulterated and Spurious Drugs and Medicines, approved 29th June,* 1848. *The Report of the Committee on the Registration of Births, Marriages and Deaths*, by Dr. Griscom, is chiefly an invocation to the profession, for the purpose of arousing the attention of the State Governments to the value and necessity of a general adoption of the measure of registration. Reference is made to the fact of registration laws being in full force in the States of Massachusetts and New York, and of steps having been taken in some others to give effect to the recommendation of the Association.

Dr. N. S. Davis, on behalf of the *Committee on Indigenous Medical Botany*, made a report, prefaced with some observations setting forth: 1. The necessity of searching diligently for new and more efficient agents in the Materia Medica. 2. The advantage, on the score both of duty and policy, of studying "the general science of botany," with a view of neutralising the influence of

Thompsonian and " Botanic " doctors; and, 3. The greater security, in the use of indigenous vegetable remedies, against adulteration and fraudulent substitutes. He communicates the pleasant information, that, recently, the profession has been favoured with a very complete catalogue of the medicinal plants of New York, by Dr. C. A. Lee. Dr. S. W. Williams and Dr. F. P. Porcher, the members of the committee from Massachusetts and South Carolina, also placed in the hands of the Chairman manuscript reports, containing very full lists of the medical plants of these states, arranged according to their natural order. The body of the report consists of a particular account of such articles as the time of the committee enabled them satisfactorily to investigate. These are: 1. RUMEX: *Water Dock, or Yellow Dock* ; 2. LYCOPUS VIRGINICUS: *Bugle weed;* 3. HAMAMELIS VIRGINICA: *Witch Hazel* ; 4. CIMICIFUGA RACEMOSA: *Actaea Racemosa, Black Cohosh.* We shall probably return to this report, ere long.

The concluding *Reports* are, on the *Number of Practitioners of Medicine in Virginia, also in Delaware and Massachusetts.* To these succeed papers, entitled, *A New Feature in the Anatomical Structure of the Genito-Urinary Organs, not hitherto described,* By Gurdon Buck, Jr., M.D. Illustrated with a Plate. Also, *Ophthalmitis Postfebrilis* ; and *A Catalogue of the Officers and Permanent Members of the American Medical Association.*

Some of the members of the Association are displaced from their homes in quite an arbitrary fashion by the Committee of Publication. Thus, for instance, Drs. Bell, Biddle, and Bond, of Philadelphia, are made denizens of Lancaster, in the Catalogue of permanent members. The Committee seem to be undecided about the use of the definite article in the " Contents " of the volume. For the most part, we read " Report of the Committee," &c. Sometimes, however, it is "Report of Committee." So of the prefixes of "Number." In one place it is " Report on the Number," &c.; in another, " Report of the Number ;" and again, " Report of Number," &c. Uniformity in such matters is desirable, were it only for the sake of accuracy, to say nothing of euphony.

Upon the whole, although this volume of Transactions is not calculated to excite national vanity, it evinces a longing and a promise for a higher and better state of things; and as such, we hail its appearance with pleasure, and recommend it to general perusal.

